# Health care, social, educational, and employment needs of individuals affected by rare diseases and their families in Catalonia, Spain

**DOI:** 10.1186/s13023-026-04281-x

**Published:** 2026-02-28

**Authors:** Encarna Calmaestra-Carrillo, Raul Vernet, Josep Torrent-Farnell, Iolanda Arbiol-Rodríguez, Àngels Cardona Cardona, Jennifer Grau-Sánchez, Misericòrdia Carles-Lavila, Núria Codern-Bové

**Affiliations:** 1 Research Group on Complex Health Diagnoses and Interventions from Occupation and Care (OCCARE), EUIT Centre Universitari, Universitat Autònoma de Barcelona, Terrassa, Spain; 2 Faculty of Nursing, Rovira i Virgili University, Campus Terres de l’Ebre, Tortosa, Spain; 3 Plataforma Malalties Minoritàries, Fundació Dr.Torrent-Farnell, Barcelona, Catalonia Spain; 4 ÀreaQ. Evaluation and Qualitative Research, Barcelona, Catalonia Spain; 5 Economic Department, Universitat Rovira i Virgili, Tarragona, Catalonia Spain; 6 Research Centre on Economics and Sustainability (ECO-SOS), Universitat Rovira i Virgili, Tarragona, Catalonia Spain; 7 EUIT Centre Universitari, Universitat Autònoma de Barcelona, C/de la Riba, Terrassa, 90. 08221 Spain

**Keywords:** Rare diseases, Supportive care needs, Psycho-social support, Health care, Public health system, Educational needs assessment

## Abstract

**Background:**

Over 7,000 rare diseases affect about 30 million people in the European Union. Living with a rare disease requires interdisciplinary care and multiple services from public systems. It is essential to have updated information about individuals affected and their families, as their needs and experiences may have evolved due to the transformations and improvements in this field over the past 15 years. This study aims to describe the perceived needs of individuals affected by rare diseases and their families, regarding healthcare, social, educational, and employment systems.

**Methods:**

A cross-sectional study was conducted using an online survey. The sample included 291 participants, comprising individuals with a rare disease or their family members responding on their behalf due to cognitive or physical difficulties. The survey included questions about sociodemographic data, diagnostic process, healthcare and rehabilitation services, psychological and social support, financial needs, and requirements in school and work settings. Data was obtained between December 2021 and February 2022 in Catalonia (Spain). Statistical analyses were conducted using the *R* programming language and software environment.

**Results:**

Forty-five percent of participants reported a diagnostic process lasting one to two years. Forty-eight percent were satisfied with the support received from professionals during the diagnostic process. All participants expressed a need for health care, rehabilitation, psychological, or social support, citing insufficient current resources. Nearly half received financial assistance, but it was deemed inadequate due to high disease-related expenses. Educational needs were reported by 31–66% of participants, including psychoeducational and psychomotor services. Regarding employment, 73% required remote work options.

**Conclusions:**

Delayed diagnosis and unmet healthcare, social, educational, and employment needs persist. Understanding the needs of patients with rare diseases is essential for an appropriate response from public systems. Recommendations include emphasising complete diagnosis and early assessment, and intervention plans for affected individuals and their families. Complex interventions that consider health, psychological, social, educational, and employment aspects are needed, with coordinated efforts focused on both the patients and their families.

## Background

Rare diseases (RD), also known as low-prevalence disorders, encompass a broad and heterogeneous group of complex and often incurable clinical conditions. In Europe, a disease is considered rare when it affects fewer than five in 10,000 individuals, according to the Orphanet definition [1]. Globally, an estimated 7,000–8,000 RD have been described, most with onset in childhood and many of genetic origin [2–4]. In the European Union, about 30 million people are affected by RD. In Spain and Catalonia, the figures are approximately three million and 350,000 individuals, respectively [2–4].

The low prevalence of RD often results in limited awareness and understanding among both the general population and healthcare professionals. Improving care for people living with RD is therefore considered both a public health challenge and a human rights priority in Europe [5, 6].

Living with an RD diagnosis is often a complex and challenging experience for affected individuals and their families [7, 8]. These conditions can affect physical health, functional autonomy, social participation, and emotional well-being, diminishing quality of life and caregivers, relatives, and other people close to them [9–12]. Evidence also highlights difficulties obtaining a timely and accurate diagnosis, prolonged diagnostic pathways due to non-specific symptoms, and limited professional expertise, underscoring the need for specialised and coordinated care [7, 8].

People living with RD often require support from multiple healthcare and social services within limited timeframes, which can be difficult to coordinate [13–15]. At the same time, households may face a substantial loss of income when patients and/or family caregivers reduce working hours or take time off work (including unpaid or only partially compensated leave), which further increases the overall burden [16, 17]. The combination of low prevalence, diagnostic difficulties, and clinical complexity means that affected individuals and families have diverse and evolving needs that health and social systems struggle to address. Despite progress in recent decades, unmet needs and difficulties in accessing resources remain [18–20].

Over the past decade, policy initiatives in the European Union, including Spain and Catalonia, have sought to improve care and support for people living with RD. European Reference Networks (ERNs) were established to connect specialised centres, share expertise, and coordinate care and research [21]. In Spain, the State Reference Centre for Rare Diseases (CREER, by its initials in Spanish) provides information and guidance on available health and social resources [22]. In Catalonia, Networks of Clinical Expertise Units have been organised to strengthen care pathways for specific groups of RD [23].

Despite these advances, up-to-date evidence on the needs and experiences of people living with RD and their families remains essential to tailor services and inform policy. We therefore conducted a survey in Catalonia (Spain) to describe perceived needs related to diagnosis, healthcare, social support, education, and employment, and to inform actionable recommendations to improve services and public policies.

## Methods

### Design

A cross-sectional study was conducted using an online survey to identify the health, social, educational, and employment needs of individuals affected by rare diseases and their families in Catalonia (Spain).

### Participants

There were several inclusion criteria. First, respondents had to be individuals affected by rare diseases or their legal representative, particularly for minors or adults unable to respond to the questionnaire due to cognitive or physical disability. Additionally, participants were required to have internet access and provide informed consent. The rarity of a disease was defined based on the Orphanet classification (< 5:10,000) [1]. Participants who completed less than 85% of the survey were excluded.

Considering the overall population of individuals living with a rare disease in Catalonia, a confidence level of 95% was used to estimate the sample size, ensuring a precision level of less than 0.07 for proportion estimation. The required sample size was 210 affected individuals, but we obtained answers and analysed data from 291 participants.

### Survey development

An online survey was developed to identify the needs of individuals and families affected by rare diseases across various topics, including diagnosis, treatment, rehabilitation, social and psychological support, education, employment, services used, and satisfaction with services.

To develop the survey, an initial literature review was conducted to identify potential frameworks, surveys, scales, and items pertinent to the study’s focus. Then, three focus groups (FGs) with executive members of rare diseases associations in Catalonia discussed and verified the appropriateness of topics and items (FG1 = 10 women and 3 men; FG2 = 10 women and 1 man; FG3 = 8 women and 1 man). Participants were selected based on convenience sampling, considering their expertise in living with rare disease.

After preparing the survey, its coherence and understandability were piloted in a sample of six participants that included three experts in rare diseases and three affected individuals. The comments from the pilot testing led to minor adjustments to the survey’s layout.

The final version of the survey comprised 41 closed questions and 28 statements using a Likert scale, with values ranging from 1 (“*strongly dissatisfied*”) to 6 (“*strongly satisfied*”). The survey was structured into five sections: (i) sociodemographic questions, including age, gender and educational level of the participant; (ii) disease and diagnostic process questions about the main disease diagnosis, date of diagnosis, age at the time of diagnosis, number of health professionals involved in the diagnosis, and level of satisfaction with the support and information received during the diagnostic process; (iii) questions related to services asking for their perceived need for healthcare, rehabilitation, social and psychological services, level of satisfaction with specialised services, coordination among social service professionals, shared decision-making and professional health care; (iv) inquiries about financial needs, and (v) questions regarding service requirements in school and work settings, including level of satisfaction with coordination between health care and educational professionals, family participation in educational decisions, and support service needs at school or in the workplace.

### Survey distribution

The Platform of Rare Diseases, a public entity belonging to the Catalan Institute of Health, facilitated survey promotion and distribution. An e-mail was sent to 53 association representatives, informing them about the study’s objectives and requesting their cooperation in distributing the online survey. A total of 43 associations responded to the request and shared the link to the survey with their members by posting it on their websites or distributing it by e-mail. The survey was available online from December 2021 to February 2022.

### Data analysis

A descriptive analysis of the variables was performed. For comparisons, the Chi-square test was used if the categories were nominal. For responses collected in the form of a Likert scale, comparisons were made with the Mann-Whitney U-test. For these types of responses, the two most extreme positive categories (“*a great deal of need”* and *“a lot of need*”; “*very satisfied”* and *“satisfied*”; and “*a great deal of support”* and *“a lot of support*”) were combined. Then, the percentage of responses and the corresponding 95% confidence interval were calculated. Associations between variables with categorized values were assessed using non-parametric Kendall’s tau rank correlation. The data were analysed using *R*, a programming language and software environment for statistical computing [24].

Finally, to ensure that the article met methodological quality standards, the Strengthening the Reporting of Observational Studies in Epidemiology checklist was used [25].

### Ethical considerations

The study was conducted following the principles of the Declaration of Helsinki and was approved by the Research Ethics Committee of the Foundation University Institute for Primary Health Care Research Jordi Gol i Gurina of Catalonia, (IDIAPJGol, 21/194-P).

## Results

A total of 291 individuals participated in the survey: 57.7% (168) were people diagnosed with a rare disease, 40.9% (119) were family members, and 1.4% (4) were legal guardians or friends who answered on behalf of the affected individual. The affected persons were 62.5% (182) women, 36.8% (107) men, and 0.7% (2) non-binary. The affected persons’ mean age was 37 years, and the 31–50 age group was the most prevalent. In terms of education, 41.1% (115) had a university degree (Table 1).

**Table 1:** Socio-Demographics Characteristics

**Respondent**	*n* = 291 (%)
Affected person	168 (57.7)
Parent	101 (34.7)
Grandparent	2 (0.7)
Partner	16 (5.5)
Guardian/friend	4 (1.4)
**Gender of the affected person**	*n* = 291 (%)
Male	107 (36.8)
Female	182 (62.5)
Non-binary	2 (0.7)
**Age of the affected person**	*n* = 291 (%)
1–15	61 (21.0)
16–30	47 (16.1)
31–50	102 (35.0)
51–64	61 (21.0)
> 65	20 (6.9)
Mean age	37 age
**Studies of the affected person**	*n* = 280 (%)
University degree	115 (41.1%)
Certificate of higher education	56 (20.0)
Vocational education and training	36 (12.9)
Certificate of secondary education	3 (1.1)
Primary education	58 (20.7)
Pre-school education	3 (1.1)
Without studies	7 (2.5)
Other	2 (0.7)

Regarding the diagnosis, 87 distinct rare diseases were reported. Following the ERN classification of rare diseases [21], the most common diagnostic group was rare skin diseases (ERN-Skin), representing 32.3% (94) of cases (Table 2). Due to the overrepresentation of this group, a comparative analysis was performed to identify possible differences in sociodemographic and study variables between this group and the other diagnoses. The analysis showed no significant differences between the groups except for resource use.

**Table 2:** Diagnosis according to ERN

ERN (European reference networks)	*n* = 291 (%)
** BOND**: Rare bone and mineral conditions	13 (4.5%)
** CRANIO**: Rare and or complex craniofacial anomalies	6 (2.1%)
** EpiCARE**: Rare and complex epilepsies	2 (0.7%)
** ERKNet**: Congenital nephrotic syndrome	17 (5.8%)
** ERN EYE**: Rare eye diseases	20 (6.9%)
** ERN LUNG**: Rare respiratory diseases	24 (8.2%)
** ERN-RND**: European reference networks – Rare neurological diseases	30 (10.3%)
** ERN-SKIN**: Rare skin diseases	94 (32.3%)
** EURO-NMD**: Europe – Neuromuscular diseases	24 (8.2%)
** EuroBloodNet**: Sickle cell disease	1 (0.3%)
** GUARD-HEART**: Rare and complex diseases of the heart in adult and paediatric patients	4 (1.4%)
** ITHACA**: Rare developmental disorders	30 (10.3%)
** MetabERN**: Rare metabolic disorders	15 (5.2%)
** RARE**-Liver: Rare liver diseases	1 (0.3%)
** ReCONNET**: Rare and complex connective tissue and musculoskeletal diseases	4 (1.4%)
** RITA**: Rare primary immunodeficiency, auto inflammatory, and autoimmune diseases	4 (1.4%)
** VASCERN**: Rare vascular diseases	1 (0.3%)
** NO NAME**	1 (0.3%)

### Diagnostic process, support needs, and satisfaction regarding services

Of the participants, 40.2% (117) received a definitive diagnosis in less than 1 year, with 22.7% (66) diagnosed within 3–12 months. Diagnostic delay was also reported: 27.5% (80) indicated that the diagnostic process took between 1 and 5 years, 9.3% (27) between 5 and 10 years, 15.5% (45) more than 10 years and 7.6% (22) did not remember. A statistically significant correlation was found between age and diagnostic delay (*n* = 269; *p* < 0.001), indicating shorter delays among younger individuals (Table 3).

**Table 3:** Diagnostic process^1^

**Time to diagnosis**			*n* = 291 (%)
Prenatal-neonatal diagnosis			23 (7.9)
> 1 < = 3 months			28 (9.6)
> 3 < = 12 months			66 (22.7)
> 1 < = 2 years			38 (13.1)
> 2 < = 5 years			42 (14.4)
> 5 < = 10 years			27 (9.3)
> 10 < = 20 years			19 (6.5)
> 20 years			26 (8.9)
Do not remember			22 (7.6)
**Age - Time of diagnosis relationship** ^2^			*n* = 269 (%)
	**< 2 years**	**2 < = 10 years**	**> 10 years**
<=15 years	43 (74.1)	15 (25.9)	0 (0)
16 < = 30 years	27 (58.7)	14 (30.4)	5 (10.9)
> 30 years	86 (52.4)	39 (23.8)	40 (24.4)
**Number of professionals involved in diagnosis**			*n* = 291 (%)
30 or more			14 (4.8)
Between 15 and < 30			15 (5.2)
Between 9 and < 15			25 (8.6)
Between 6 and < 9			22 (7.6)
Between 3 and < 6			109 (37.5)
Less 3			76 (26.1)
None			6 (2.1)
Do not know			24 (8.2)
**Assessment of support received from professionals**			*n* = 289 (%)
Very satisfied			54 (18.6)
Satisfied			85 (29.4)
Indifferent			49 (17.0)
Dissatisfied			46 (15.9)
Very dissatisfied			55 (19.0)

^1^Percentages are calculated within each section-specific denominator (n)

^2^“Do not remember” is not included. Kendall correlation 0.203. p-value 0.0003

Regarding the number of professionals involved in the diagnostic process, 26.1% (76) consulted fewer than three professionals, 37.5% (109) consulted three to five professionals, and 10.0% (29) needed more than 15 professionals. In terms of the support received by professionals throughout the diagnostic process, 48.1% (139) were satisfied or very satisfied with the support received (*n* = 289) (Table 3).

### Needs for treatment, rehabilitation, social and psychological support, and satisfaction with services

Nursing services were used 1–2 times every six months by 31.3% (41) of respondents. Family medicine or paediatrics was used 1–2 times every six months by 45.7% (74).

A notable portion of the sample indicated that they either lacked access to certain health services or required more services than they were currently receiving. Specifically, 34.7% (68) expressed a desire for more genetic counselling, 16.7% (22) indicated a need for increased nursing services, and 8.0% (13) reported needing more services from family medicine or paediatrics (Table 4).

**Table 4:** Services and resources required for treatment, rehabilitation and support^1^

**HEALTH SERVICES**							
	**Nursing service** *n* = 131 (%)	**Family medicine/paediatrics** *n* = 162 (%)			**Genetic counselling** *n* = 196(%)		
I need more care	22 (16.7)		13 (8.0)			68 (34.7)	
Once a year or sporadically	37 (28.2)		50 (30.9)			89 (45.4)	
1–2 times every 6 months	41 (31.3)		74 (45.7)			29 (14.8)	
1–3 times a month	24 (18.3)		23 (14.2)			0	
1–3 times a week	7 (5.3)		2 (1.2)			0	
I don’t know	0		0			10 (5.1)	
**REHABILITATION SERVICES**							
	**Rehabilitation** *n* = 145 (%)	**Nutrition** *n* = 133 (%)		**Speech therapy** *n* = 66 (%)	**Physiotherapy** *n* = 170 (%)		**CDIAP**^2^ *n* = 47 (%)
I don’t have access but would like to	52 (35.9)	67 (50.4)		27 (40.9)	63 (37.1)		23 (48.9)
Once a year or sporadically	30 (20.7)	23 (17.3)		7 (10.6)	22 (12.9)		8 (17.0)
1–2 times every 6 months	24 (16.6)	37 (27.8)		3 (4.5)	36 (21.1)		5 (10.6)
1–3 times a month	23 (15.9)	5 (3.8)		6 (9.1)	38 (22.4)		7 (14.9)
1–3 times a week	16 (11.0)	1 (0.8)		23 (34.9)	11 (6.5)		4 (8.6)
**SOCIAL SUPPORT OR PSYCHOLOGICAL CARE SERVICES**							
	**Social work services** *n* = 106 (%)		**Support groups** *n* = 148 (%)			**Psychological consultations** *n* = 189 (%)	
I don’t have access but would like to	49 (46.2)		71 (48.0)			62 (32.8)	
Once a year or sporadically	39 (36.8)		24 (16.2)			23 (12.2)	
1–2 times every 6 months	13 (12.3)		15 (10.1)			14 (7.4)	
1–3 times a month	1 (0.9)		23 (15.6)			34 (18.0)	
1–3 times a week	4 (3.9)		15 (10.1)			56 (29.7)	
**PERCEIVED SATISFACTION**							
	**Specialized services** *n* = 277 (%)	**Professional coordination** *n* = 255 (%)		**Shared decision-making** *n* = 254 (%)	**Healthcare professionals** *n* = 264 (%)		**Social system professionals** *n* = 142 (%)
Very satisfied	79 (28.5)	49 (19.2)		51 (20.1)	49 (18.6)		13 (9.2)
Satisfied	89 (32.1)	69 (27.1)		62 (24.4)	96 (36.4)		36 (25.4)
Indifferent	44 (15.9)	29 (11.4)		45 (17.7)	46 (17.4)		39 (27.5)
Not satisfied	31 (11.2)	53 (20.8)		48 (18.9)	47 (17.8)		32 (22.5)
Very dissatisfied	34 (12.3)	55 (21.6)		48 (18.9)	26 (9.8)		22 (15.4)

^1^ Percentages are based on the number of participants who reported needing each service (n); therefore, denominators differ across services

^2^CDIAP: Child development and early care centres

Regarding rehabilitation, 11.0% (16) of participants received care 1–3 times week, while 20.7% (30) received care once a year or sporadically. Many respondents reported unmet access needs for several services. The highest proportions reporting “no access but would like to” were for nutrition consultations 50.4% (67), and services from child development and early care centres (CDIAP) 48.9% (23). Unmet access needs were also frequent for speech therapy consultations 40.9% (27), physiotherapy services 37.1% (63), and rehabilitation services 35.9% (52) (Table 4).

For social and psychological support, 46.2% (49) reported not having access to social work services but would like to, while 36.8% (39) used social work services once a year or sporadically. For psychological consultations, 18.0% (34) reported attending 1–3 times a month, whereas 32.8% (62) reported not having access but would like to. For support groups, 15.6% (23) attended 1–3 times a month and 48.0% (71) reported not having access but would like to. Overall, the high proportions reporting “no access but would like to” across multiple services highlight a substantial gap in care provision (Table 4).

Over 60.6% (168) of participants reported being satisfied or very satisfied with specialised services, and 55.0% (145) with the care provided by professionals. Satisfaction was lower for coordination among professionals 46.3% (118), shared decision-making 44.5% (113), and professional care within the social system 34.6% (49) (Table 4).

### Financial needs and employment and satisfaction regarding financial support

The results indicated that living with a rare disease has an impact on the family’s financial and employment situation. Among the participants, 45.0% (130) reported receiving financial assistance related to the disease, mainly in the form of government aid, disability benefits, or other public allowances. In terms of employment impact, 36.5% (105) reported that a family member stopped working to provide care. Among those reporting employment impact (*n* = 105), 48.6% (51) stopped working entirely and 49.5% (52) reduced working hours (Table 5).

**Table 5:** Financial and employment consequences of low-prevalence diseases/financial support needs

**FINANCIAL AND EMPLOYMENT NEEDS**	
**Need financial help**	*n* = 289 (%)
Yes	130 (45.0)
No	159 (55.0)
**Family member stopped working to provide care**	*n* = 288 (%)
Yes	105 (36.5)
No	183 (63.5)
**Type of employment change among those reporting Yes**	*n* = 105 (%)
Stopped working entirely	51 (48.8)
Reduced working hours	54 (51.4)
**PARTICIPANTS’ PERCEPTION OF THEIR FINANCIAL NEEDS**	
**Pharmacological treatment**	*n* = 180 (%)
I can pay	151 (83.9)
I can’t pay	29 (16.1)
**House accessibility improvements**	*n* = 152(%)
I can pay	78 (51.3)
I can’t pay	74 (48.7)
**Home care professional**	*n* = 108 (%)
I can pay	39 (36.1)
I can’t pay	69 (63.9)
**Psychological support**	*n* = 169 (%)
I can pay	81 (47.9)
I can’t pay	88 (52.1)
**Assistive products**	*n* = 181 (%)
I can pay	145 (80.1)
I can’t pay	36 (19.9)
**Diapers or similar**	*n* = 55 (%)
I can pay	46 (83.6)
I can’t pay	9 (16.4)
**Rehabilitation treatment**	*n* = 177 (%)
I can pay	97 (54.8)
I can’t pay	80 (45.2)
**Technology**	*n* = 133 (%)
I can pay	100 (75.2)
I can’t pay	33 (24.8)
**LEVEL OF SATISFACTION WITH FINANCIAL SUPPORT**	*n* = 237 (%)
Very dissatisfied	110 (46.4)
Somewhat dissatisfied	55 (23.2)
Indifferent	41 (17.3)
Satisfied	25 (10.6)
Very satisfied	6 (2.5)

Participants identified several expenses as difficult or impossible to afford: 63.9% (69) reported difficulties hiring a home care professional, 52.1% (88) paying for psychological support, 48.7% (74) making home accessibility improvements, and 45.2% (80) in covering rehabilitation treatments. The level of satisfaction with the financial support received was low, with 46.4% (110) and 23.2% (55) of respondents, respectively, declaring themselves very dissatisfied or somewhat dissatisfied (Table 5).

### Needs in school or work settings and satisfaction with services

Despite the reported needs in the school setting, 60.4% (61) of families expressed being satisfied or very satisfied with the support provided by the teaching staff. Likewise, 29.4% (27) of families were satisfied or very satisfied with the coordination between educational professionals and healthcare services, 46.7% (42) were satisfied or very satisfied with shared decision-making between the teaching team and parents regarding schooling decisions for their children (Table 6).

**Table 6:** Needs of people affected by low-prevalence diseases in school and work settings^1^

**Perception of the most necessary services and resources in a school setting**	*n* (%)				
Psychological or psycho-pedagogical support (*n* = 71)	47 (66.2)				
Psychomotor therapy (*n* = 59)	38 (64.4)				
Individual classroom aide (*n* = 61)	39 (63.9)				
Specialised support teacher (*n* = 58)	37 (63.8)				
Setting adaptations (*n* = 62)	36 (58.1)				
Speech therapist (*n* = 54)	31 (57.4)				
Technological adjustments (*n* = 63)	33 (52.4)				
Nursing care (*n* = 66)	21 (31.8)				
**Perception of the most necessary services and resources in a work setting**	n (%)				
Remote work (*n* = 71)	52 (73.2)				
Setting adaptations (*n* = 65)	37 (57.0)				
Technological adjustments (*n* = 64)	30 (46.9)				
Nursing care (*n* = 71)	18 (25.4)				
	**Very satisfied**	**Satisfied**	**Indifferent**	**Not satisfied**	**Very dissatisfied**
Satisfaction with support child receives at school (*n* = 101)	31 (30.7)	30 (29.7)	16 (15.8)	16 (15.8)	8 (8.0)
Satisfaction with coordination between health professionals and school (*n* = 92)	11 (12.0)	16 (17.4)	29 (31.5)	20 (21.7)	16 (17.4)
Satisfaction with shared decision-making between teacher teams and family (*n* = 90)	17 (18.9)	25 (27.8)	27 (30.0)	12 (13.3)	9 (10.0)
Satisfaction with support received from occupational health services (*n* = 111)	10 (9.0)	25 (22.5)	23 (20.7)	20 (18.1)	33 (29.7)

^1^Percentages are based on item-specific denominators (n), which vary depending on item applicability

In the work setting, the most frequently reported need was remote work options 73.2% (52). Better workplace adaptations were required by 57.0% (37), and technological adaptations by 46.9% (30). In addition, 25.4% (18) reported needing nursing care in the workplace, and 31.5% (35) reported being satisfied or very satisfied with the support received from occupational health services (Table 6).

## Discussion

These results show that having a RD has a major impact on the daily lives of affected individuals and their families. In addition, participants reported diagnostic delays and unmet needs for healthcare, social support, education, and employment assistance for people with rare diseases and their families.

Notably, we observed a shorter time to diagnosis compared with previous studies [26, 27]. While our study was not designed to identify the underlying causes, this improvement may reflect recent advances in RD care in Spain—particularly in Catalonia—including the expansion of newborn screening programmes, broader access to genetic/genomic testing in routine practice, and the development of specialised networks that facilitate referral and coordinated care [28]. In addition, the lower the age of the affected individual, the shorter the delay in diagnosis. These findings are consistent with the cross-sectional study conducted in Spain 2016–2017 [9], but contrast with previous studies, indicating that 24% of participants took more than five years to receive a diagnosis [27, 28]. However, a wide range of participants in our study experienced a diagnostic process lasting more than one year, highlighting the need to continue addressing this challenge.

Despite these improvements, substantial diagnostic delays persist for a proportion of people living with a RD, with some individuals waiting more than 10 years for a definitive diagnosis. Although our study cannot identify the causes of this delay, Spanish registry-based evidence suggests it is often linked to complex diagnostic pathways requiring multiple specialist consultations, the need to travel or be referred to other provinces/regions to access expertise, and long waiting times for referral after the first medical visit, particularly in certain disease groups (e.g., nervous system disorders) [29, 30].Delaying a formal diagnosis can take a psychological toll on both patients and their families [7]. Diagnostic delays must be reduced to improve recovery prospects and better support the needs that arise during the diagnostic process [18]. We found a moderate level of satisfaction with the support received. In line with previous research [31], there is room for improvement in terms of satisfaction with the support received during the diagnostic process. Psychological support can make a significant difference to the quality of life and empowerment of patients who receive a diagnosis of a rare disease. This support, along with knowledge about the disease and possible treatments, can provide resources to cope with the challenges of living with a rare disease [32]. In this context, and to ensure the benefits of a comprehensive biopsychosocial approach, nurses could play a key role in monitoring and coordinating communication with those affected [33].

Beyond diagnosis, lower satisfaction with interprofessional coordination in this study underscores the need to strengthen care coordination. In Spain, advanced nursing competencies in rare diseases include case management and coordination across levels of care [34]. Prior studies report nurse-integrated models such as nurse involvement in genetic day clinics [35], coordinated multidisciplinary clinics for ultra-rare conditions (e.g., Alström syndrome) [36], and structured transition programmes from paediatric to adult services [37]. Case-management pilots (e.g., INNOVCare) also highlight cross-sector coordination for complex rare diseases [38]. These examples provide practical options to embed nursing coordination in multidisciplinary services, informing future development in Catalonia.

Our findings reveal substantial perceived barriers to accessing essential services, as reflected in the high proportion of participants who wanted to use these services but were unable to do so. This gap between needs and service availability suggests limitations in current provision and warrants attention in service planning. The results of our study are consistent with Depping et al. [31], who highlight the need to improve healthcare systems, as most affected individuals have unmet psychological, social, personal, health, and information needs. Strategies such as the use of screening tools, involvement of expert patients in multidisciplinary care, and the adoption of integrated, social, and holistic approaches to person-centred care may help address these gaps [39, 40].

Participants reported high dissatisfaction with the financial support received. Consistent with previous studies in other contexts [14, 16, 41], families perceived public financial contributions as insufficient to cover their needs, particularly those associated with medical treatment. Furthermore, the dependence evolving from these types of diseases often requires personal assistance, which primary caregivers usually provide, significantly impacting their social, professional, and educational lives. Regarding the paediatric population, for instance, many parents have to resort to several adaptation strategies and integrate complex protocols into their family’s daily routine to manage their child’s illness [42]. The participants perceived dissatisfaction with financial aid could be linked to their satisfaction (or lack thereof) with social service professionals. Only 34% reported satisfaction or great satisfaction, possibly influenced by the fact that most aid is distributed through the social system.

Although our questionnaire did not specifically explore the reasons for dissatisfaction with the financial burden, evidence suggests that the financial burden on families living with a rare disease is largely due to high direct and indirect costs, including non-medical expenses, informal care, and lost productivity, along with limitations in social protection and available financial aid. A study on the socioeconomic costs of rare diseases, describes considerable out-of-pocket expenses and a significant risk of financial overburdening for affected households, which may help explain the dissatisfaction with the financial burden reported by some participants in our survey [43].

In terms of education, families reported that psychological or psycho-pedagogical care, psychomotor services, individual classroom aides, and/or specialised support teachers are necessary resources and services. Despite these demands, our study highlights the high level of satisfaction expressed in this area, possibly due to support services currently available to educational teams and families coping with students who have special educational needs. Furthermore, associations have played a role in providing training to the educational community. However, caution is needed when interpreting these results because the impact of these diseases is highly variable, thereby increasing diverse support service requirements. Due to their significant biological and motor complexity, certain diseases require more nursing care, while others involve more teaching support to ascertain the child’s or adolescent’s autonomy in school. All these services or resources should be guaranteed to ensure equity in the schooling of children and adolescents. Attention problems, reduced motivation for achievement, decreased social competence, decreased autonomy, and low self-esteem are educational aspects that ultimately affect the quality of life of rare disease patients and their families [44, 45].

In the employment context, individuals affected by rare diseases noted the need for schedule flexibility, setting adaptations, and technological adjustments, with a large majority expressing the need for remote work opportunities. This finding may relate to experiences during the COVID-19 pandemic, which have enabled exploring successful ways of working remotely. In general, people have realised that work tasks can effectively be performed remotely, making the implementation of working-from-home options a priority today.

For future research, the analysis of unmet needs by ERNs and the adoption of mixed-methods approaches would be valuable to gain deeper insights into the specific requirements and circumstances of different rare disease groups [46]. Furthermore, in line with European recommendations, it is necessary to increase translational and social research aimed at deepening our understanding of the necessities for health and rehabilitation services, social support, and psychological care for specific disease groups and age ranges.

This study has limitations that should be acknowledged. Firstly, the study is based on individuals affected by rare diseases who are linked to associations or the Rare Diseases Platform. Additionally, the questionnaires were answered by self-report. Finally, comparison with previous studies is not possible because there are no validated instruments specific to the scope of this study. These limitations may have led to response bias or inaccuracies in assessing needs. The strengths of the study lie in the sample size and the use of real-world data.

## Conclusions

This study provides evidence of and insight into the current needs perceived by individuals affected by a rare disease and their families, including the difficulties and limitations they face in their daily lives in terms of health care, education, and employment.

A diagnostic delay was clearly present, and only half of the individuals reported satisfaction with the support provided during the diagnostic process. Therefore, there is great potential for improvement. Access to required services was perceived as limited, and financial assistance was deemed insufficient. A perception prevailed that both support and services must be expanded for children in schools and adults in the workplace. Identifying these needs provides a starting point for analysing and discussing current policies in health care, social support, education, and employment.

It is essential to recognise that each rare disease has distinctive characteristics. However, many share similar challenges in terms of diagnosis, access to treatment, health care, social and financial support, as well as the impact on the quality of life of patients and their families. For this reason, to facilitate generalisability, the differences and similarities between various rare diseases must be considered, while simultaneously involving all stakeholders to effectively address common challenges.

## Implications for policy and practice

In accordance with the findings and following the suggestions outlined in the “Rare 2030 Foresight in Rare Disease Policy” report, we offer a series of recommendations for enhancing procedures [18]. These recommendations were presented jointly with the Rare Diseases Platform to the Health Commission of the Parliament of Catalonia in March 2023.

During the diagnostic process:

- Promote policies that ensure access to accurate and timely diagnosis, reducing diagnostic delays.
- Keep up research efforts to ensure that everyone receives a formal diagnosis to the fullest extent possible.
- Strengthen the holistic approach to care throughout the diagnostic process.
- Improve the quality of information provided during the diagnostic process.

Needs for services and resources related to treatment, rehabilitation, and support:

- Enhance translational and social research efforts to gain deeper insights into the requirements for healthcare services, rehabilitation, social assistance, and psychological treatment tailored to specific disease clusters and age demographics, with the objective of reassessing existing criteria for care, treatment, rehabilitation, and support.
- Improve collaboration areas among experts from various departments.
- Improve the engagement of individuals affected by a condition and their families in treatment, rehabilitation, or support processes through the utilisation of shared decision-making models.

Economic support needs:

- Increase financial support to ensure rehabilitation services and support for the individual and their family.

Needs for services in the educational and employment domains:

- Strengthen psychological, psycho-pedagogical, psychomotor, caregiver, and support teacher services in schools.
- Consider revising employment regulations to encourage opportunities for working remotely.

Holistic needs:

- Guarantee equal opportunities, education, and job access for affected individuals with rare diseases by incorporating person-centred approaches throughout their lives, alongside ensuring fair treatment, social security, and inclusion.

## Data Availability

The datasets used and analysed during the current study are available from the corresponding author on reasonable request.
